# Perceptions of the community leaders on adolescents’ HIV status: cross sectional study in Mulanje, Malawi

**DOI:** 10.11604/pamj.2020.37.71.24484

**Published:** 2020-09-17

**Authors:** Chancy Skenard Chimatiro, Precious Luwidzyi Hajison, Adamson Sinjani Muula

**Affiliations:** 1 School of Public Health and Family Medicine, College of Medicine, University of Malawi, Blantyre, Malawi,; 2 Africa Center of Excellence in Public Health and Herbal Medicine, University of Malawi, College of Medicine, Blantyre, Malawi,; 3 PreLuHa Consult, Namiwawa Street, Newroard Location, P.O Box 703 Zomba, Malawi

**Keywords:** HIV, adolescents, community leaders

## Abstract

**Introduction:**

the spread of HIV among adolescents requires effective interventions as new infections are high in this cohort globally. We explored perception of community leaders on the prevalence of adolescent´s HIV in Mulanje, Malawi.

**Methods:**

focus group discussion (n=11) and in-depth interviews (n=15) were conducted with community leaders in all Traditional Authorities in Mulanje district, Malawi. The interviews were audio recorded and transcribed. Data were analysed using thematic content approach.

**Results:**

the perceptions of community leaders on the HIV prevalence among adolescents fall into three groups: lack of access to health services in rural areas, cultural beliefs and social practices. Unavailability of condoms and youth centres were perceived to contribute to lack of access to health services. On harmful cultural beliefs, study participants observed that initiation ceremonies and prolonged wedding dances at night were contributing to HIV spread. Several issues were reported on social issues including poverty, illicit drug and substance abuse, long distances to school, modern technologies and peer pressure.

**Conclusion:**

there are many factors perceived to contribute towards high HIV prevalence among adolescents. Our study suggests urgent need for the country to sustainably address key harmful cultural and social practices that potentially increase adolescents´ vulnerability to HIV.

## Introduction

Reducing the further spread of Human Immunodeficiency Virus (HIV) among adolescents is a global challenge. This study was conducted in order to understand the perception of community leaders on what may be contributing to the HIV prevalence among adolescents in Mulanje district, Malawi. Globally, adolescents make up to 16% of the total population [1]. About 85% of the adolescent population lives in low and middle income countries [2]. Adolescence is a transitional stage from childhood to adulthood with specific health and developmental needs and several opportunities and risks [3]. During this stage of growth, many high risk and unhealthy behaviours may be initiated by the adolescents such as smoking, alcohol drinking, substance use and misuse as well as unprotected sexual intercourse [4]. The high risk and unhealthy behaviours practiced by the adolescents can lead to short to medium-term health problems such as unplanned pregnancies or long term illnesses such as cancers, heart diseases on the way into adulthood.

It is estimated that over 830 adolescents are infected with the HIV every day worldwide [5] and new cases are high in girls than boys due to multiple vulnerabilities including biology, poverty and sexual abuse with most of these infection occurring in the sub-Saharan region with over 45% of the world´s HIV infections [6]. The 2018 data by the United Nations Children´s Education Fund (UNICEF) indicate that 85% of adolescents living with the virus are in sub-Saharan Africa [7], of which 58% are girls [8]. Poverty, cultural practices, early sexual debut and sexual abuse are associated with high HIV burden among adolescents in this region [9]. In Malawi, there are over 4.1 million adolescents representing 23.7% of the country´s total population [10]. About 83.3% of adolescent girls and 84.1% of adolescent boys live in rural areas [11]. Early sexual activity is high with around 15% of young women and 18% of young men (aged 15-24) reporting having sex before the age of 15 [12, 13]. The 2015-16 Malawi Demographic and Health Survey reported the overall HIV prevalence of 2.1% among adolescents [14] which is higher in girls (3.3%) than boys (1%). Several reasons why adolescent girls are more vulnerable to HIV than boys have been documented elsewhere [15].

Mulanje is one of the districts in Malawi with adolescents HIV prevalence of 7.8% [16]. Many families in the district are economically poor and adolescents are involved in unprotected transactional sex in search of money [17]. Several interventions have been channeled towards adolescents in the district. Among these interventions is the promotion of Sexual and Reproductive Health (SRH) practices such as Youth Friendly Reproductive Health Services (YFRHS) [18]. According to our literature search, there is lack of data on how community leaders perceive adolescents' HIV status in Mulanje. Yet, understanding the perception of community leaders on the HIV prevalence among adolescents can assist policy makers and implementing partners to come up with the interventions that can reduce further the spread of the virus and improve the health status within this age group.

## Methods

This was a cross section, qualitative study of which Focus Group Discussions (FGDs) and In-depth Interviews (IIs) were conducted. Data collection was done between the months of October and December 2018. Mulanje district has six Traditional Authorities (TAs) namely: Juma, Mabuka, Chikumbu, Njema, Mthiramanja and Nkanda with many of its inhabitants being Lomwe. The IIs and FGDs were conducted in all six TAs. The study participants included Traditional Authorities (TAs), sub-traditional leaders, chiefs, religious leaders, initiation counselors and leaders of ethnic groups (*eni mbumba*). In total, the study had 110 participants of which 15 participated in IIs and 94 in FGDs. The sample size was reached after saturation point when no new answers were coming out from the study participants during both IIs and FGDs.

We used purposive sampling technique to recruit all leaders who participated in both IIs and FGDs from all TAs. We reached the saturation point after conducting 11 FGDs and 15 IIs. The IIs included TAs, Sub-TAs, religious leaders, ward councillors and initiation counselors. The participants in FGDs were Group Village Headmen (GVH), village headmen, initiation councilors, religious leaders and leaders of ethnic groups. We targeted those who had held leadership positions and dwelt in the area for a period of not less than a year. This gave us an in-depth understanding of the perception of the community leaders as the targeted participants had enough experience with what happens within their communities.

The study recruited three research assistants in addition to the principal researcher (CSC). The research assistants were trained for a period of one week to be familiar with the study protocol and data collection tools. We used two voice recorders during each of the interview to secure the data in case one develops fault. The field team shared responsibility while in the community. During FGDs, the principal researcher was responsible for asking guidelines questions, note taking and controlling the proceedings. One assistant was responsible for managing the recorders. Finally, the two assistants were also responsible for taking demographic characteristics of the study participants. The IIs were conducted through face by face interviews between one field worker and the respondent.

The recorded voices were listened to several times for familiarisation. We used the local Chichewa language in the transcription of data which was later translated in English language by an expert from the Department of Languages, Chancellor College, University of Malawi. The researcher checked the original voice recording in local language Chichewa against English translated texts for accuracy one at a time. Further, the other authors also checked the translated texts, both Chichewa and English transcriptions to ensure that there were no discrepancies. The emerged themes were grouped and assigned codes. We then developed themes from the transcribed data. We assigned the codes under the theme it supported. We finally analysed the data manually.

We obtained ethical approval from the College of Medicine Research and Ethics Committee (COMREC). The permission to conduct the study in Mulanje was granted by the District Commissioner (DC). We explained the purpose of the study to the eligible participants. The Participant Information Sheet was read to all eligible participants to understand the important of the study and why they should take part before signing the consent. The written consent or witnessed consent with a thumb print from those who were unable to read was obtained. We used numbers instead of personal identifiers for the research participants. The participants were allowed to choose a place where to conduct interviews. Participants were allowed to discontinue the interviews anytime they felt so.

## Results

In total, we conducted 11 FGDs and 15 IIs. Table 1 below summarises the socio demographic characteristics of the study participants. The findings were grouped into three main themes. The themes include lack of access to health services, cultural belief and social practices. Further, each theme had sub-themes.

**Table 1:** socio-demographic characteristics of the study participants

Characteristic	Female	Male	Total
**Marital status**			
Married	40	50	90
Single	6	0	6
Divorced/Separated	11	3	14
Total	57	53	110
**Highest education level**			
None	7	6	13
Primary	35	35	70
Secondary	11	16	27
Total	53	57	110
**Religion**			
Roman Catholic	10	11	21
Church of Central African Presbyterian	12	9	21
Islam	2	8	10
Other Christian churches	31	27	58
Total	55	55	110
**Leadership Positions**			
Traditional Authorities	2	3	5
Ward Councillors		2	3
Village Headmen	9	18	27
Sheikh/Church leader	7	9	16
Family ethnic elders (*eni mbumba*)	9	15	24
Initiation Counsellors	4	2	6
Others	22	6	28
Total	55	55	110

### Lack of access to health services

**Unavailability of condoms in rural areas:** the community leaders indicated that lack of condom supply in rural areas put lives of many adolescents at a risk of contracting HIV. A study participants reported the following: *“The health centre is far away from here where the young ones can get condoms as such they don´t have anything for protection when they are having sex intercourse which increases their risk to HIV”* (A female respondent No 8, FGD 5).

*“Aaaaah achimwene (my brother) you know some youth are open! As a village head I have been receiving complaints from youth about unavailability of condoms within our locality which has put lives of our youth at risk”* (A male participant, IIs No 3).

**Lack of youth centres in rural areas:** some of the community leaders mentioned lack of youth centres in rural areas where adolescents receive health information and meet role models as another perceived factor that contribute towards HIV prevalence. One of the community leaders reported that: *“We normally hear that in some areas there are youth centres where adolescents receive health information and also get condoms”* (A male respondent 7, FGD 1). Another community leader reported that; *“There is no role models here who can motivate adolescents to resist from sexual activities”* (A female participant, IIs No 14).

**Cultural belief:** some cultural beliefs were mentioned to be among the contributing factors perceived by the community leaders.

**Initiation ceremonies:** initiation ceremonies were mentioned as one of cultural practices that subject adolescents to HIV. It was reported that the cultural practices during these ceremonies are performed by both girls and boys and increase their desire for sex. The majority of community leaders mentioned “dancing on the door” (*Kubvina pachitseko*) as one of the cultural practices which has increase adolescents´ risk to HIV. Kubvina pa Chitseko is a cultural practice performed a day before initiated girls come out of the initiation camp. Girls are dressed in very short skirts, with beads around their waists and necks while breasts are uncovered for everyone to see. Elderly women carry a door on their head while an adolescent is on top dancing, displaying what they have learnt at the initiation camp. This is done in presence of everyone including adolescent boys and men. This practice was reported as attracting men to have sex with those girls who were dancing well once out of initiation ceremony. The community leader reported that: *“We have this practice here called Kubvina pa Chitseko (dancing on the door), which is practiced a day before young girls come out of initiation ceremony. This practice subject many girls to engage in early sexual intercourse because they feel that they are now fully grown and can sleep with any man irrespective of their age”* (A female participant No 9, FGD 8). Another respondent reported that: *“We are still doing it in secrecy, we are only telling you because we hope you will hide our identity. Kubvina pa chitseko subjects many girls here to early sexual activities as girls are spotted by men during the event and when they go out of the initiation camp they usually try to practice what they have learnt there”* (A male respondent, IIs 15).

The majority of community leaders especially women mentioned *Masosoto* (Pulling of labium minora to grow using natural herb oil made from Nsatsi) as one of the cultural practices that arouse early sexual intercourse among adolescent girls. According to the community leaders who mentioned it, *Masosoto* is a practice whereby older women teach adolescents while in initiation camp to use castor oil (*Nsatsi*) to pull out their labium minora to grow. They believe that only women with long labium minora attract and keep their families for long. A woman community leader reported that: *“Young girls are taught some practices that increase their desire to have sexual intercourse once out of the initiation camp like Masosoto thereby contracting HIV”* (A female respondent, IIs 2).

Another respondent reported that: *“Girls are not told to practice sexual intercourse once out of the initiation camp but I believe some practices like Masosoto encourages them to do so which subject them to HIV”* (A female participant 5, FGD 8). The community leaders felt that natural herbs such as “Nthubulo” given to the adolescents when they are coming out of initiation camp increase their sexual desire. A community leader reported that: *“The herbs given to these young boys and girls (meaning adolescents) when coming out of initiation camps increases desire for sexual intercourse. This forces them to indulge in unprotected sexual intercourse thereby getting the virus”* (A male respondent, IIs 7).

**Prolonged traditional dances during weddings:** the community leaders also mentioned of the prolonged traditional dances during weddings as another perceived factor that put lives of many adolescents at high risk of contracting HIV. A community leader reported that: *“The prolonged wedding preparations dances (mganda) during nights subjects many adolescents to unhealthy habits as they take advantage of that to engage in sexual activities”* (A male participant 3, FGD 1). Another community leader reported that: *“Most of the adolescents here sleep in their own apartments (gowelo). During night wedding preparation dances, they pretend as if they are going to sleep yet they wake up when we are asleep to get girls for sexual intercourse from these dances which put them at a risk of contracting HIV”* (A male respondent 6, IIs 3).

**Social factors and practices:** some social factors were identified as contributing to the high risk of HIV among adolescents.

**Poverty:** the majority of community leaders mentioned poverty as a factor that put adolescents at risk of HIV. One of the community leaders reported that: *“Many families here are poor and they don´t provide enough resources to their children which forces them to go into relationship with older men who provide them with their needs, end result is that the adolescents contract HIV”* (A male respondent, IIs 13). Another community leader mentioned that: *“Poverty of the parents force adolescents to indulge in bad habits as they lack some basic needs such as soap or even exercise books, as a result they try to find someone who can provide those items ending up exchanging with sex intercourse”* (A female participant 3, FGD 9).

**Drug and substance abuse:** many community leaders reported of the substance abuse especially alcohol consumption and marijuana smoking (*chamba*) as one of the perceived factors contributing to the HIV incidence among adolescents. A community leader reported that: *“Nowadays most of these adolescents smoke marijuana and drink beer, while drunk they are involved in many bad habits including sexual intercourse hence ending up contracting HIV”* (A male participant 5, FGD 3). Another community leader reported that: *“Adolescents especially boys are drinking alcohol too much and once drunk they involve in unhealthy behaviour with older women ending up contracting HIV”* (A female respondent, IIs 3).

**Lack of girls´ hostels and long distances to schools:** some community leaders mentioned the lack of institutional girls´ hostels and long distances to the community secondary schools as one of the perceived factors. A village head reported that: *“The community secondary school is very far from here and there is no hostels for the girls. So even if they can indulge in sexual activities and reach home late nobody can question them”* (A female participant 6, FGD 3). Another community leader indicated that: *“I think on their way back from school they are involved in many unhealthy activities including coerced sexual intercourse which put them on high risk of acquiring HIV as there are no girls hostels at the secondary school”* (A male respondent, IIs 4).

**Modern technologies:** access to high technological cellphones among adolescents was reported as another factor by majority of community leaders. One of the community leaders reported that: *“The naked images transferred using modern cellphones in the hands of adolescents increases their unprotected sexual activities”* (A female respondent, IIs 2). Another community leader reported that: *“I am always concerned to see the adolescents with modern phones. They use these phones to browse pornography and they end up trying what they have seen risking themselves to HIV infection”* (A male participant 8, FGD 3). Video shows were also mentioned to influence adolescents to indulge in high risk behaviours such as early sexual intercourse thereby contracting HIV. A community leader reported that: *“Video show rooms doesn´t have age limit so many young people learn immoral behaviours there which endanger their lives”* (A male participant 2, FGD 6).

**Peer pressure:** some community leaders mentioned peer pressure among adolescents as another perceived factor that increases their risk of contracting HIV. A religious leader reported that: *“The problem of HIV among adolescents cannot end because most of them follow what their friends do especially girls without protecting themselves”* (A male participant 2, FGD 10). Another community leader reported that: *“The adolescents are fond of copying what their friends do even if it is unhealthy including sexual activities behaviours. This act subjects many of them in this area to HIV infection”* (A female respondent, IIs 5).

## Discussion

Our study was aimed at assessing the perceptions of community leaders on the HIV prevalence among adolescents in Mulanje, Malawi. Adolescents undergo many challenges in their life as they grow into adulthood. These challenges, if not addressed effectively have short, medium and long-term adverse consequences. Results from this study show that community leaders associate adolescent´s HIV with many factors ranging from the lack of access to health services, cultural practices, social economic challenges and harmful practices. The findings of this current study agree with another study also done in Mulanje, Malawi where harmful cultural practices were also mentioned [19]. Limited access to health services including availability of condoms among adolescents in rural areas is a challenge as this study has indicated. Condom use among adolescents is an important intervention that can reduce HIV infection in this age group. However, with the lack of condoms in the community setting as was reported in the study, it was not clear to us whether one can still access them at the health facilities due to long distances. Many villages in rural areas are far away from these health facilities which makes it difficult for the adolescents to access condoms. Condoms may also not be available in the retail shops, and where available, may be unaffordable to adolescents. Long distances to health facilities as a barrier to health services accessibility was also reported somewhere [20]. The Malawi National HIV strategy included condom use as one of the preventive interventions [21]. However, the country should strengthen its health systems to ensure that condoms are available in rural areas by using community health structures such as Health Surveillance Assistants (HSAs) and Community Condom Distributors (CCDs).

The presence of YFRHS and clubs in rural areas is another way of improving HIV awareness among adolescents. The Malawi Government started implementing YFHS in 2007 [22], unfortunately, the coverage is slow. The scarcity of condoms and unavailability of youth centres where adolescents can meet peer educators were mentioned by the community leaders as perceived factors increasing the vulnerability of adolescents to HIV. These findings agree with what was reported in another study done in the northern part of Malawi where the importance of involving peer educators who were role models proved to be effective [23]. Cultural practices and beliefs form an important element of every community. However, some cultural practices and beliefs which are harmful need to be discouraged [24]. This study shows that harmful cultural practices are being performed in some parts of the country which puts the lives of adolescents at risk of acquiring HIV. In addition, the current study has shown that *Kubvina pa chitseko, Masosoto* and *Nthubulo* are some of the cultural practices performed during initiation ceremonies that are erotic. The findings of this study are similar with what was reported by Kalipeni E *et al*. in the study done in Thyolo and Mulanje-Malawi where they also reported that cultural practices performed during initiation ceremonies increase the vulnerability of adolescents to STIs including HIV [25]. We suggest that even if these dances can be performed in absence of boys and men, adolescent girls will want to try sexual intercourse to prove that they are fully grown. It is worth noting that community leaders in Mulanje are reported to be at the forefront restricting harmful cultural practices and beliefs [26]. Based on this, it is essential that the community leaders should restrict such activities to safeguard the lives of adolescents especially girls. Further, our study has reported that adolescents are also given herbs when coming out of the initiation camps which is perceived to increase their sexual desire. We suggest that the use of herbs with potential aphrodisiac properties be avoided. This practice should be abolished by the community leaders as it subjects adolescents to high risk of contracting HIV.

The community leaders also perceived that social practices put adolescents at risk of HIV. Alcoholism and drug abuse were mentioned as a social practice performed by adolescents that increase their risk of contracting and spreading HIV. Although different study methodology were used, similar findings were also reported in other studies done in Malawi by Page and Muula [27, 28]. Furthermore, lack of girls´ hostels and long distances to the community secondary schools is another perceived factor that increases HIV transmission among adolescents as they indulge in sexual activities on their way back home as reported in our study. Long distances between destinations may be detrimental for the adolescents (especially girls) as they cannot protect themselves even when sex intercourse is coerced. This finding was also reported in the final evaluation of the joint programme on adolescent girls in Malawi by Munthali *et al*. that long distances to school subject many adolescents to the risk of contracting HIV [29]. We suggest that relevant authorities should consider provision of institutional girls´ hostels to ensure that they are protected from coerced sexual intercourses. The availability of modern cellphones in the hands of youth was perceived as having increased HIV infection as their desire for sexual intercourse increases with what they normally see on the internet such as *“pornographic“* films and sexting [30]. Modern cellphones have various apps and sites that can be accessed at any time and give adolescents ample time for peer sexual data transfers. Additionally, pop-ups from some sites are erotic and as such, with the inquisitiveness of the adolescents they click on those links which lead them to sexual sites. This was also reported in another study on a collaborative path to comprehensive adolescent sexual and reproductive health and rights in our time [31].

Some adolescents do not have modern cellphones, therefore, others indulge in sexual activities with older men in order to get money to buy one and other needs. Parents should take a leading role in monitoring and informing their children especially adolescents on sexual intercourse issues to reducing habits of sexting. We suggest change of mind set on parental side so that they can freely discuss sexual issues with adolescents. This will assist adolescents to have accurate and correct information relating to sexual intercourse and prevent further spread of HIV in this age group. Peer pressure to match friends was mentioned as one of the perceptions that increases HIV infection among adolescents. In another setting with different design, peer pressure was also mentioned in a study done in Tanzania [32] where it was reported that girls would like to be seen like others around them which is similar with what this study found. However, not all activities performed by peers are of desirable outcomes as this study has suggested. Some of the activities increase the risk of acquiring HIV among adolescents. Therefore, the availability of YFRHS and youth clubs in rural areas can assist adolescents in avoiding HIV acquisition and transmission.

## Conclusion

There are many factors perceived by the community leaders as the contributing factors towards HIV prevalence among adolescents in Mulanje, Malawi. Our study suggests urgent need for Malawi as a country and Mulanje in particular as a district to address all harmful cultural and social practices that increase adolescents´ vulnerability to HIV. Adolescents should have access to health services including ensuring continuous availability of condoms in rural areas. Stakeholders and community leaders should collaborate in their work if the fight against HIV is to be won. Further, we suggest that parents should open up to discuss sexual intercourse related issues with their children. This should not be seen as a taboo within the communities. Moreover, stakeholders implementing HIV activities in Mulanje should focus much on scaling up evidence-based activities that can reduce further spread of HIV among the adolescents.

### What is known about this topic

- Many interventions have been implemented in low and middle income countries including Malawi focusing on reducing the spread of HIV among adolescents;
- The sub-Saharan region is most affected and a home to over 85% of adolescents living with the HIV globally.

### What this study adds

- Lack of access to health services like availability of condoms in rural areas increases adolescents´ vulnerability to HIV;
- Cultural beliefs such as initiation ceremonies and prolonged wedding dances as well as social practices like poverty, illicit drug and substance abuse, long distances to school, modern technologies and peer pressure are among the contributing factors towards HIV prevalence among adolescents;
- HIV among adolescents is viewed as a challenge that requires an urgent attention in the communities.
